# The Chronic Indigence of Our Hospitals, with Some Suggestions for Its Remedy

**Published:** 1892-04-23

**Authors:** B. Burford Rawlings


### The Institutional Workshop.

### THE CHRONIC INDIGENCE OF OUR HOSPITALS, WITH SOME SUGGESTIONS FOR ITS REMEDY.

By B. Burford Rawlings.

### IV—WHAT MIGHT BE.

Seeing that an overwhelming weight of opinion has declared itself in favour of maintaining the voluntary character of medical relief, the question naturally and pertinently arises: What means can the hospitals employ in order to remedy their poverty and their deficiencies by their own unaided action? It is unfortunate that most people should regard voluntary and casual as interchangeable terms, because from that confusion of idea springs a chief source of mischief. A contribution surely may be voluntary and yet in no sense casual, and further, it is impossible that any system of voluntary support for continuous and indispensable work, such as hospital work is, can be reliable and satisfactory if it do not cease to be casual. The essence of a method of voluntary support is that people should be brought to regard a share in it as not only a duty to be performed but a privilege to be enjoyed, and if only the public mind fully realised all that is involved in the maintenance of our hospitals as benevolent institutions, where the poor in sickness obtain a community of advantages with the rich, they would not allow them to change their character for want of such trifling individual help as is needed to render them safe. It is too often forgotten that there is a very marked difference between the work and responsibilites of our hospitals and the work and responsibilities of any other philanthropic undertaking whatever. In the case alike of an asylum or a soup kitchen, the relief afforded may be strictly adjusted to the means. There is no obligation to do more than the contributions supplied are sufficient for. But hospitals can make no such nice balance of means and work. Day and night their doors stand open, and for any one of them to refuse to treat an applicant because it had no funds would be denounced as an outrage.

It is useless, therefore, for critics and economists to blame the hospital which gets into debt, always assuming that it has striven to keep out of it, because as long as the means supplied are unequal to the cost of the work demanded, debt is inevitable. We have to deal with two now notorious facts —one is that only a very small minority of the population contributes towards the maintenance of our hospitals, and the other, that a minute annual subscription contributed by all well-to-do householders alike would give the hospitals enough and to spare. The Lord Chancellor, who not long ago expressed himself appalled at certain figures put forward by the writer, showing the proportion of contributors to population, has since announced that he has verified the statement. There is no doubt in the minds of those who look into the matter concerning the neglect the hospitals suffer, especially at the hands of the ultra rich, and if further testimony were needed, we have it in the published account of wills, representing £200,000 of personalty and upwards proved during the last three months. The total of the personalty has reached £6,569,738, and of this enormous sum medical charity has been allotted £50, represented by sums of £25 each to two county dispensaries.

Seeing the very obvious nature of the claim the hospitals present, it is no wonder that Lord Halsbury went on to express an opinion that the institutions had only to make their wants better known to get them attended to. Alas! that a deduction so reasonable should receive such scant confirmation. By letter, by advertisement, by speech, the voices of the hospitals go up unceasingly, and the public expresses itself aweary of their importunity. If the institutions travel outside the limited circle of their own supporters, it has been already shown that they find mankind deaf, and seeing that they fail to obtain a recognition of such direct obligation as is incurred when a rich man or woman obtains admission for a dependent and insists upon the all-sufficiency of a "letter," how can it be hoped that they will succeed in penetrating the apathy of the prosperous crowd to whom hospital work comes in so impalpable a form that even selfishness is not aroused?

It is the opinion of most hospital financiers that the difficulties in the way of raising funds by the old-fashioned methods of solicitation and appeal are steadily increasing. Begging, though, for the most unimpeachable objects, is full of discouragement. But granting that each individual institution continues to find means to exist, what prospect is there of solving the problem how to render the work complete? If everything is to be accomplished by voluntary means, it is indispensable that the area from which subscriptions are drawn should be greatly enlarged, and this is precisely what it seems cannot be brought about by fragmentary and competitive efforts. Each hospital, if able to hold its own, does so only by unrelaxing vigilance and labour, and by expending everything which comes to hand. To carry the position in front demands a combined assault.

The cause our hospitals represent, put forward as a whole, would be irresistible; but it is imperilled by a want of unison among the institutions. Vexed questions, not wholly financial, but with a financial side, call for settlement, and it is indispensable if the work is to be made as perfect and as comprehensive as it ought, that our hospital system should be something more than a mere fortuitous congregation of institutions. It would be easy to act in concert without surrendering the best part of individuality, and on the other hand hospitals, if they value their independence, will be wise to take warning by the signs of the times. The truest friends of the hospitals are not they who persistently close their eyes to obvious defects and think they have proved everything that is necessary, because they can show that this or that particular hospital is a model of efficiency. The perfection of individual workers is quite consistent with the existence of both deficiency and excess in the body. And is it unworthy of the intelligence of the age to regard the medical relief of our sick poor, and the diffusion of medical knowledge as something capable of being properly and comprehensively dealt with by an ill-assorted bevy of independent institutions without one connecting tie, with no provision whatever for either conference or co-operation, and competing one against the other for alms wherewith to carry on their self-apportioned labours? Common sense rebels at the contention that things are best as they are, and it may be taken for granted that if voluntary medical relief is to survive, the erection of a central body, a sort of Hospital Parliament, is inevitable. What have legitimate hospitals to fear? We can imagine trepidation in certain quarters, but what can be the objection on the part of the managers of reputable institutions to an authority born of their own flesh and blood, not meddlesome, but sympathetic, and composed of men of like aims with themselves? The erection of a central council ought to be welcome, if only because it would help towards a code of laws under which responsible hospitals would be glad to live. However divergent the views taken of the effect of Hospital Sunday upon the finances of the institutions, it would be ungrateful to deny that much of the influence it has exercised upon their administration has been salutary.

Given a truly representative body, it would prove comparatively easy to enlist a co-operation fairly denied to rival and multitudinous appeals. Dean Swift thought that the greatest benefactor of his race would be the man who could make two blades of grass to grow where one had grown before. If we compare the area of obligation to our hospitals with the sparse sheaves charity gleans upon the widest fields, it does not seem a stupendous task to raise two subscriptions where now the yield is one. If the householders, for instance, whose names appear in the "Court Directory," and the cream of the suburban population, who number 100,000 and upwards, people who pay from £80 to £500 apiece by way of rent and rates, and everyone of whom is indebted to our hospitals for the medical aid he and his family are able to secure, could be induced as a class to make some acknowledgment of obligation, say, by an annual subscription of £4 or £5 for each household, not another appeal need be issued. Besides these, there are the large trading companies and employers of labour whose subscriptions now amount to only a fraction of the expense involved in the treatment of patients they introduce, and the great landlords. None would be more likely than the last to respond in a fitting manner, when they felt assured that their contributions would save them a never-ceasing solicitation. Some years ago the writer suggested how potent the co-operation of the life assurance companies might be rendered. Many of these great corporations have made more or less liberal grants to individual hospitals, and it may be believed that if the proposal for a more active assistance reached the companies from the representatives of the art and practice of medicine in the metropolis, and was the outcome of a well-considered scheme embracing all necessities, it would receive favourable attention. Let us suppose the companies consented to collect for the hospitals a voluntary contribution from policy-holders of threepence in the pound of premiums payable in London. Allowing for a fifty per cent. margin of refusals, the annual product would considerably exceed £100,000 Where agreed to, the gift would represent an addition of just half-a-crown for every ten pounds of premium paid, and what, we may pertinently ask, would those premiums have been but for the work of the voluntary hospitals? Perhaps with an enlightened appreciation of the circumstances, the life companies would have established hospitals and maintained them at their own cost, just as the fire companies at one time supported the Fire Brigade. In any case, whether they had thus atoned for the nation's neglect, or had been content to share it, the cost must have been ultimately out of the pockets of the insurers, if, indeed, in the second alternative life insurance had been practicable at all.

It is strictly consistent with a system in which poverty is exalted to a virtue that the patients of our hospitals should not be permitted to do anything in return for the benefits they receive, and it is natural that the objection to contributions from patients should come from the honorary medical staff. It is only recently that any hospital of position has been able to accept a payment for the food of certain classes of in-patients. The rule that no return in any form is to be accepted from out-patients is maintained with scarcely an exception. There is no recognition of such varying means and circumstances as arise within the limits of eligibility. All must be reduced to a dead level of indigence, and to many a patient compelled by stress of circumstances to seek the relief of our hospitals it must appear almost barbarous to refuse him a liberty to acknowledge, if not to discharge, his indebtedness.

We will not do the physicians and surgeons the injustice of supposing their objections are merely selfish. A patient who can pay a consultant's fee is necessarily ineligible. But the members of the staff are bound to certain methods, and they are convinced that to insist upon hospital relief being free is a way of protecting the profession at large. Now, when we are dealing with a body of patients numbering a million and upwards, we want something better than an arbitrary and unaccommodating rule. If it be certain that one half the patients cannot pay anything at all, it is equally certain that in the aggregate the other half could pay a great deal. Assuming half a million of out-patients to give three attendances apiece, and to contribute an average sum of one shilling a visit, a sum of no less than £75,000 would be provided annually from that source alone.

It may be observed parenthetically that if the effect of this were to send the shilling patients to the private doctor, then a great deal would have been accomplished towards remedying the congested state of the out-patients' departments; but the objection will no doubt take quite another shape. It will be said that the general practitioners would be robbed of patients. Scarcely, as the persons we are considering are already attending at the hospitals. But the general practitioner has every right to be protected, and his position in regard to our hospitals is one which might well be modified. On the one hand it would be beneficial if the general practitioner were enabled to keep in touch with the hospital practice, and thus to extend the advantages of a post-graduate system of education, and on the other, the out-patients' departments provide ample work for a very largely augmented staff.

At present we have a small body of consultant physicians and surgeons, the flower of the profession, and according to the custom of the particular hospital, they are themselves required to treat the patients, or, if assistance be given, it is by young men of the school, at best just qualified. Neither course is quite satisfactory. Under the first system, a single medical officer must prescribe for fifty or more patients during an afternoon—an ordeal trying to himself and equally so to the unfortunate patients, many of whom must wait for hours before being accorded a brief attention; while in the second, we have the cases of patients diagnosed and treated by inexperienced youths. Except upon the ground of indigence, what valid objection can there be to the creation of a sufficient number of paid appointments tenable for a fixed period and open to *bona fide* general practitioners of, say, three years' standing? There is room for 200 of such appointments, while the £75,000 collected from the patients would suffice to pay each of these gentlemen £150 annually for an attendance of three afternoons weekly, and leave a handsome balance towards the drug bill. A payment of this amount, plus the undoubted advantages they would obtain from the hospital connection, would be enough to secure the best men of their class and to ensure that feeling of obligation to the hospital and its authorities which is now often wanting. The details of a scheme of the kind would, of course, need to be carefully considered, and it would be indispensable that the full scientific power of the hospital, as represented by its consultant staff, should be available in every case of doubt and difficulty.

This question of payment by patients is in all likelihood destined to prove a powerful factor in the solution of the chronic financial difficulty of our hospitals. We want an enlightened and kindly system of inquiry into the circumstances of each individual patient with a view to his occupying his proper place in the list of contributories. In the case of out-patients, the payment might range from nothing to one or even two shillings each attendance, and in the case of in-patients, from nothing to twenty-five shillings per week. As a rule, a bread-winner would be free, but what wisdom is there in refusing to recognise that a husband in full work may be just as well able to contribute towards the maintenance of his wife in hospital as he is to support her at home, or that a father may be called upon without hardship to help towards the keep of his child? With this reform once fairly started, every hospital for children ought to see its way to raising a very large proportion of its expenditure among the patients' friends, and every other hospital, more particulaly those that are special, might look to command from this source a substantial increase of reliable income. The National Hospital (Queen Square), which employs ample safeguards against the admission of unsuitable patients, has set apart wards for "contributing" patients, and in that way receives upwards of £2,000 per annum from a class of sufferers as deserving of help as any, but who are able to meet three-fourths of the actual cost of their maintenance while in the wards.

When we come to gather up the several suggestions made in the course of these papers we find upon the one hand a proposal to add to the hospital expenditure by a direct and systematic remuneration of the medical staff and upon the other various methods of enlarging the usefulness of the hospitals and diminishing their dependence upon casual contributions. We have started with the *Lancet's* estimate of a present total expenditure of £606,000, but as the calculation was made for Sunday Fund purposes, we think it likely that St. Bartholomew's and St. Thomas's, neither of which participates, are excluded from the calculation. The total expenditure of the whole of the metropolitan voluntary hospitals is in all probability nearly £700 000, and if we add to this the £130,000 we have named approximately as the sum needed to remunerate the staff, and appropriate £50,000 which might be saved by the absorption of unnecessary hospitals to the maintenance of 1,000 additional beds for convalescents, we have a sum of £830,000 as the annual cost of maintenance. The estimate for paying the staff is arrived at thus: 150 physicians and surgeons for in-patients, average 50 beds a-piece, with a fee of £400 per annum each, £60,000; 150 physicians and surgeons for out-patients, at £250 each, £37,500; 200 assistants to physicians and surgeons for out-patients at £150, £30,000; a total of £127,500.

Towards this expenditure we may reckon (roughly) the sums provided by endowments at £250,000, the lists of annual subscriptions at £100,000, the Sunday and Saturday Funds at £60,000, patients' contributions (in and out) at £150,000, making a total of £560,000. An average annual subscription of five guineas each from one-half the 100,000 householders in the Court Directories who now contribute nothing would supply the balance required, leaving the other indicated new sources of income, and all sums derivable from donations and legacies, available for expenditure upon capital account, or for investment by the several recipients.

These proposals practically leave untouched both the administrative independence and the voluntary character of the support upon which hospital authorities set so great value. All they involve is the creation of a representative body, charged with consultative functions, capable of approaching with success certain classes hitherto inaccessible to individual appeals, and charged with the distribution of the £250,000 derivable from these sources upon an equitable basis of division—the simpler the better. No doubt the ability to raise so large a sum by this means will be called in question, but when we reflect upon what has been accomplished in many directions by individual energy, there need be no misgiving. Every successful institution is a monument to hard work, and no form of genius is better adapted to the needs of hospital finance than the capacity for taking trouble.

The case for the hospitals has never been presented with sufficient boldness, because it has never yet had the advantage of being put forward in its entirety. There is an obligation to our medical institutions which cannot be urged in respect of other forms of charitable work, whose benefits are limited to those actually relieved. The influence of our hospitals is conterminous with our civilization, and everyone who has called in a doctor has consciously or unconsciously taken tithe of these beneficent institutions. Let this point, as well as the duty we owe to the sick poor, be carefully insisted upon by a capable organisation, and the result will not be doubtful. But necessary above all things is that full recognition of an identity of interests which leads societies, like individuals, to genuine and whole-hearted co-operation. If there be one lesson more than another to be read in the signs of the times we live in, it is the importance, nay, the indispensability, of combination. No undertaking, however great, can afford to stand isolated from its fellows. Examine what class of successful enterprise you will, you find the units subordinating their individual liberties to promote the common good, and in that way attaining a power denied to isolated efforts. It is not enough our hospitals should deserve success; for the sake of our civilization and our humanity they must be able to command it.

